# Direct interaction between avian pathogenic *Escherichia coli* and H9N2 avian influenza virus promotes bacterial adhesion during their infections

**DOI:** 10.1128/spectrum.02978-25

**Published:** 2026-06-15

**Authors:** Yue Li, Yulu Xue, Yuji Quan, Peng Chen, Xiangkai Zhuge, Tao Qin, Sujuan Chen, Daxin Peng, Xiufan Liu

**Affiliations:** 1 College of Veterinary Medicine, Yangzhou University 38043, Yangzhou, China; 2 School of Public Health, Nantong University 66479, Nantong, China; 3 Jiangsu Co-Innovation Center for the Prevention and Control of Important Animal Infectious Disease and Zoonoses, Yangzhou University 38043, Yangzhou, China; 4 Joint International Research Laboratory of Agriculture and Agri-Product Safety, the Ministry of Education of China, Yangzhou University 38043, Yangzhou, China; 5 Jiangsu Research Centre of Engineering and Technology for Prevention and Control of Poultry Disease, Yangzhou University 38043, Yangzhou, China; Universita degli Studi di Bari Aldo Moro, Bari, Italy

**Keywords:** H9N2 avian influenza virus, avian pathogenic *Escherichia coli*, complex, adhesion, pathogenesis

## Abstract

H9N2 subtype avian influenza virus (AIV) and avian pathogenic *Escherichia coli* (APEC) are prevalent pathogens in the poultry industry, and their co-infection results in synergistic pathogenicity in chickens and significant economic losses. However, the direct interactions between H9N2 AIV and APEC during infection remain unclear. We preincubated H9N2 AIV particles with APEC and found that H9N2 AIV could bind directly to the surface of APEC. The H9N2 AIV-APEC complex enhanced bacterial adhesion to chicken embryo fibroblasts and chicken macrophages (HD11), which could be inhibited by antiserum against H9N2 AIV. The chicken challenge experiment demonstrated that the complex caused significant reductions in body weight, increased bacterial loading in various organs, and resulted in more severe histopathological lesions than APEC alone. However, the complex did not affect virus replication and shedding but elicited a robust hemagglutination inhibition antibody response when compared to H9N2 AIV alone. Additionally, we found that a 3 h interaction between H9N2 AIV and APEC in the complex led to significant morphological changes in the viral envelope, ultimately resulting in the inhibition of virus replication *in vivo*. These findings suggest that the direct interaction between H9N2 AIV and APEC is more conducive to bacterial infection, providing new insights into the synergistic interaction between bacteria and influenza viruses during the early stages of co-infection with H9N2 AIV and APEC.

**IMPORTANCE:**

H9N2 subtype avian influenza virus and avian pathogenic *Escherichia coli* are common pathogens in the poultry industry. Their co-infection causes more severe harm and leads to substantial economic losses for the industry, yet the direct interaction between them remained unclear previously. Studies have found that they can bind directly; the formed complex facilitates bacterial invasion of chicken hosts and results in more severe damage. This uncovers the key reason for the greater harm of co-infection, providing a new direction for the prevention and control of such diseases in the poultry industry.

## INTRODUCTION

The H9N2 avian influenza virus (AIV) has been circulating in poultry in China for several decades (1). Although considered a low-pathogenic AIV and easily overlooked by humans, it exhibits highly efficient transmission capabilities and has caused great economic losses (2, 3). The H9N2 virus occasionally infects mammals and humans, and also serves as a reservoir for internal genes for other subtypes of AIVs, including H5N6, H7N9, H10N8, and H3N8 (4), thereby posing a significant public health risk. In poultry, simple infection with H9N2 AIV typically does not manifest obvious clinical symptoms or lead to mortality; however, high mortality rates are associated with co-infection with bacteria or other viruses (5–8). In China, H9N2 AIV is currently controlled mainly by inactivated vaccines, which can mitigate economic losses to some extent. H9N2 AIV can still be isolated from immunized chickens, which makes the virus still highly prevalent in China (9, 10).

Avian pathogenic *Escherichia coli* (APEC) is the most common bacterial pathogen in poultry and causes many diseases in commercial poultry (11, 12). H9N2 AIV and APEC exhibit synergistic pathogenic effects, with substantial evidence indicating that the H9N2 virus causes chickens to be more susceptible to secondary APEC infections (13). In terms of mechanism, H9N2 AIV infection leads to a reduction in the expression of mucin-forming primary gel-forming mucin, endogenous trefoil factor family peptides, and tight junction proteins, resulting in severe intestinal damage and promoting the proliferation and translocation of APEC (14). H9N2 AIV infection triggers inflammation in the intestinal mucosa and stimulates the secretion and release of nitrate from the host intestinal epithelium, thus facilitating APEC proliferation (15). In addition, H9N2 AIV infection enhanced the adhesion of APEC to epithelial cells by upregulating fibronectin expression. Furthermore, the viral protein NS1 of the H9N2 virus increases fibronectin expression by activating the transforming growth factor-beta (TGF-β) pathway (16, 17).

Researchers have also observed that the influenza virus can directly form symbiotic relationships with certain bacteria to achieve mutual benefits. Specifically, H1N1 swine influenza virus can bind to the capsular sialic acid of *Streptococcus suis* serotype 2 and significantly enhance the adhesion and invasion of bacteria into epithelial cells (18). Influenza viruses can directly interact with common gram-positive and gram-negative bacteria in the human respiratory tract, enhancing bacterial colonization and invasive diseases of bacteria within the body (19). Furthermore, reciprocal interactions between the influenza virus and common bacteria in the respiratory tract not only contribute to the environmental stability of the virus but also improve its infectivity (20). The stability of *Streptococcus pneumoniae* is enhanced in the presence of the influenza virus when shed by co-infected hosts (21). Both H9N2 AIV and *E. coli* are common pathogens in the respiratory tract of poultry; however, the direct interaction between H9N2 AIV and *E. coli* remains unclear. Here, we established a co-infection model using H9N2 AIV and APEC and evaluated the effect of the H9N2 AIV-APEC complex on bacterial colonization, viral loading, and disease severity in co-infection.

## MATERIALS AND METHODS

### Bacteria strains, viruses, and cells

The APEC strains (Table 1) were isolated and identified from diseased chickens in Jiangsu Province, China. The serotypes of the isolates were identified by PCR and further confirmed by slide agglutination tests (22, 23). The presence of virulence genes *tsh*, *cvaC*, *iss*, *iroN*, *irp2,* and *iutA* was detected by PCR amplification (24). The isolated and laboratory *E. coli* strains were cultured overnight in LB medium (Sangon Biotech, China) at 37°C until mid-log phase. The bacteria were then suspended in DMEM to achieve a multiplicity of infection (MOI) of 100 for co-infection experiments. The H9N2 AIV strain A/chicken/Taixing/10/2010 (TX) was propagated in 9-day-old specific-pathogen-free (SPF) embryonic chicken eggs (25). Chicken embryoblast cells (CEF), chicken macrophage-like cells (HD11), and Madin-Darby canine kidney cells (MDCK) were cultured in Dulbecco’s modified Eagle’s medium (DMEM, HyClone, USA) supplemented with 10% fetal bovine serum (Gibco, USA) and incubated at 37°C in a humidified incubator with 5% CO_2_.

**TABLE 1:** The *E. coli* strains used in this study

Strains	Serotype	Virulence genes						Total numbers	Source
		*tsh*	*cvaC*	*iss*	*iroN*	*irp2*	*iutA*		
J11	O78	0	0	0	0	0	0	0	This study
J41-2	O78	0	0	0	0	0	0	0	This study
J36-1	O78	0	0	1	1	1	0	3	This study
J36-5	O78	0	0	1	1	1	0	3	This study
J48-1	O78	0	1	0	0	1	0	2	This study
J87-1	O1	0	0	1	0	0	0	1	This study
J37-4	O1	0	0	1	1	1	0	3	This study
J53-1	O1	1	1	1	1	1	1	6	This study
J54-2	O1	1	1	1	1	1	1	6	This study
J64-2	O1	0	1	1	1	1	1	5	This study
J92-3	O2	0	0	0	0	0	0	0	This study
J45-1	O2	1	0	1	1	1	1	5	This study
J45-2	O2	1	0	1	1	1	1	5	This study
J45-4	O2	1	0	1	1	1	1	5	This study
J76-3	O2	1	1	1	1	1	1	6	This study
DH5α	/^*a*^	0	0	0	0	0	0	0	Sangon
BL21	/	0	0	0	0	0	0	0	Sangon
Top10	/	0	0	0	0	0	0	0	Sangon

^*a*^ “/” indicates strains lacking a classical O-antigen serotype (rough strains; non-typable), including DH5α, BL21, and Top10.

### Virus concentration and purification

The virus was propagated by inoculation into chicken embryos. Allantoic fluid was collected and centrifuged at 4°C and 12,000  ×  *g* for 30 min. The resulting supernatant was collected and filtered through a 0.22 µm membrane. Subsequently, the filtrate was ultracentrifuged at 4°C and 30,000 rpm for 90 min using a 32 Ti rotor. A discontinuous sucrose gradient (20%, 40%, and 60%) was prepared in a 41 Ti centrifuge tube, and the concentrated virus solution was layered on top. After ultracentrifugation at 4°C and 30,000 rpm for 90 min, the virus band between the 40% and 60% sucrose layers was carefully collected. The collected virus was diluted 10-fold in 0.1 M PBS and then ultracentrifuged again at 4°C and 30,000 rpm for 90 min using a 32 Ti rotor. The supernatant was discarded, and the pellet was resuspended in 0.1 M PBS and allowed to dissolve overnight at 4°C. The purified virus was stored at −70°C.

### Co-sedimentation of *E. coli* and AIVs

The APEC J11 strain (serotype O78, low-pathogenic) at 10^8^ colony-forming units (CFU) in PBS was combined with 0.1 mL of H9N2 AIV TX strain (10^7.5^ TCID_50_/mL) and incubated at 37°C for 30 min. Bacterial and viral complexes were precipitated by centrifuging the mixture at 10,000 *g* for 3 min. The supernatant was discarded, and the pellets were resuspended in PBS and subjected to two additional rounds of centrifugation to remove unbound virus. To quantify the co-precipitated virus, the pellets were resuspended in 100 μL of DMEM containing 1% penicillin-streptomycin-gentamicin solution (Sangon Biotechnology, China) after the final centrifugation. The viral titer was determined by TCID_50_ measurements in MDCK cells.

### Negative staining

The co-sedimented H9N2 AIV and APEC mixture was fixed with 2.5% glutaraldehyde and placed on a 400-mesh carbon-coated copper grid. Subsequently, the complexes on the grids were stained with 1% (wt/vol) phosphotungstic acid. The samples were examined using a Tecnai T12 transmission electron microscope.

### Tissue culture adherence assays

To determine the adhesion of APEC to H9N2 AIV-infected cells, CEF or HD11 cells were cultured in 24-well plates at a density of 2 × 10^5^ cells per well and cultured overnight. Cells at confluence (80%–90%) were infected with TX at MOI of 0.1, 1, and 10. After 24 h post-infection, cells were inoculated with APEC J11 strain at an MOI of 100 and cultured with 5% CO_2_ at 37°C for an additional 2 h. For the co-infection experiments with H9N2 AIV and APEC*,* live or inactivated TX (10^5^ TCID_50_/mL) and J11 (MOI = 100) were inoculated simultaneously into confluent cell monolayers in 24-well plates and cultured with 5% CO_2_ at 37°C for 2 h. When required, the virus TX was inactivated by heat treatment at 56°C for 10 min, UV irradiation for 1 h using a 30 W UV lamp, treatment with 1‰ formaldehyde or 1‰ β-propiolactone for 12 h, respectively, before being mixed with the J11 strain. The inactivation of virus was confirmed by the TCID_50_ test, and the hemagglutination (HA) titer was determined by the HA test. For the antibody inhibition test, the cells were treated with antiserum against TX (diluted 1:64, hemagglutination inhibition [HI] titer = 6 log_2_) for 2 h after virus infection. Meanwhile, the control group was treated with an equivalent dilution of negative serum. The inoculated cells were washed thrice with PBS (pH 7.4) to remove unbound bacteria and viruses. Cell mixtures were collected by adding 700 μL/well of 0.05% trypsin, allowing them to stand for 5 min, and then adding 300 μL/well of a 5% BSA-PBS solution. The number of CFU per milliliter in the mixtures was determined by serial dilution and plating on MacConkey agar.

### Immunofluorescence analysis

The *E. coli* BL21 (DE3) strain harboring the EGFP plasmid (BL21-EGFP) was induced with IPTG during the logarithmic growth phase and cultured for an additional 4 h. Subsequently, 10^8^ CFU of BL21-EGFP alone, 10^8^ CFU of BL21-EGFP together with TX (at an MOI of 100), or the pre-incubated BL21-EGFP-TX complex (pre-incubation for 30 min) were inoculated into the confluent cell layer and incubated for 2 h. After three PBS washes, the cells were fixed with 4% paraformaldehyde and stained with DAPI at a dilution of 1:1,000. Finally, the cells were observed under a fluorescence microscope.

### Chicken challenge

Three-week-old SPF White Leghorn chickens (mean body weight 150 g–200 g, obtained from Lihua Agriculture, Zhejiang, China) were randomly divided into five groups: the APEC infection group (J11), H9N2 AIV infection group (TX), viral and bacterial co-infection group (TX+J11), virus-bacteria pre-incubation group (TX-J11 complex), and PBS group (PBS), 15 per group. Chickens were inoculated intranasally with J11 (10^8^ CFU) and/or TX (10^6^ TCID_50_/mL), as well as pre-incubated mixtures containing the same infectious amounts. Clinical symptoms and body weight were monitored until 7 days post-infection (dpi). Laryngeal and cloacal swabs were collected at 1 and 4 dpi for viral shedding analysis, while serum samples were collected at 1, 4, and 7 dpi for HI antibody detection (26).

Five chickens in each group were euthanized by cervical dislocation at 1, 4, and 7 dpi, and the heart, liver, spleen, lungs, kidneys, and trachea were collected for bacterial quantification, viral load determination, and cytokine expression analysis. Simultaneously, the spleens and bursas of three chickens from each group were weighed at 7 dpi, and the lymphoid organ index for each chicken was calculated as the ratio of organ weight to body weight multiplied by 100. Homogenization of each tissue (0.1 g) was performed by combining it with 1 mL PBS, followed by serial dilution. The resulting mixture was applied to MacConkey agar plates for colony counting. Total viral RNA was extracted using TRIzol Reagent (Vazyme Biotech, China), followed by reverse transcription to cDNA using TransScript All-in-One First-Strand cDNA Synthesis SuperMix for qPCR (TransGen Biotech, China), and real-time quantitative polymerase chain reaction with the indicated primers and probes (Table 2) using PerfectStart II Probe qPCR SuperMix UDG (TransGen Biotech, China).

**TABLE 2:** Primers used in this study

Gene	Sense primer (5′–3′)	Antisense primer (5′–3′)
M-Probe	CCTCAAAGCCGAGATC	
M	CTTCTAACCGAGGTCGAAACG	CTTTAKCCAYTCCATGAGAGC
β-Actin	GAGAAATTGTGCGTGACATCA	CCTGAACCTCTCATTGCCA
IL-1β	TCGACATCAACCAGAAGTGC	GAGCTTGTAGCCCTTGATGC
IL-6	TTCGACGAGGAGAAATGCTT	CCTTATCGTCGTTGCCAGAT
TNF-α	GCCCTTCCTGTAACCAGATG	ACACGACAGCCAAGTCAACG
iNOS	AGGCCAAACATCCTGGAGGTC	TCATAGAGACGCTGCTGCCAG
IFN-α	TCTGATGCAGCAGGTGGG	AGGGCTCTCCAGACTTCTGCT
IFN-β	AAGAGTTACACTGCCTTTGCC	CACTGTCTGCTGGTGGAGTTC
IFN-γ	GGGTGGGCCTCTTTTCTCAG	CTGCAGATCATCCACCGGAA

Additional tissues were collected at 7 dpi for histopathological examination. Tissues were fixed in 4% paraformaldehyde, embedded in paraffin, sectioned, and stained with hematoxylin and eosin. Histopathological changes were evaluated by a blinded examiner and scored semi-quantitatively using a 0–3 scale based on the severity and extent of lesions: 0, no significant lesions; 1, mild lesions (focal or minimal inflammatory infiltration, slight tissue damage); 2, moderate lesions (multifocal inflammatory infiltration, moderate tissue damage); 3, severe lesions (diffuse inflammatory infiltration, extensive tissue necrosis or structural destruction) (27).

### Thermal stability analysis

H9N2 AIV TX strain (10^7.5^ TCID_50_/mL) and APEC J11 strain (10^8^ CFU) were combined in 1 mL of PBS and incubated at 25°C, 37°C, and 42°C for 3 h. The mixture was centrifuged at 10,000 × *g* for 3 min, and the supernatant was collected for virus titer determination using hemagglutination and TCID_50_ assays. In addition, TX (10^7.5^ TCID_50_/mL) was mixed with J11 (10^8^ CFU), 0.1 mg/mL of *E. coli* lipopolysaccharide (LPS), or PBS control, and then incubated at 37°C for 3 h. The mixture was further examined by electron microscopy. The TX-J11 complex or TX alone after incubation at 37°C for 3 h was used for chicken intranasal challenge experiments, with three per group.

### Statistical methods

All *in vitro* experiments were performed in three independent replicates unless otherwise stated. Data were analyzed using GraphPad Prism software (version 9.0). Data are expressed as mean ± standard error of the mean (SEM). Two-group comparisons were made using the Student’s *t*-test. To compare multiple groups, one-way analysis of variance (ANOVA) followed by Tukey’s multiple comparison test was performed. Statistical significance was set at *P* < 0.05.

## RESULTS

### H9N2 AIV infection or co-incubation promotes the adhesion of APEC to avian-origin cells

**Fig 1:**
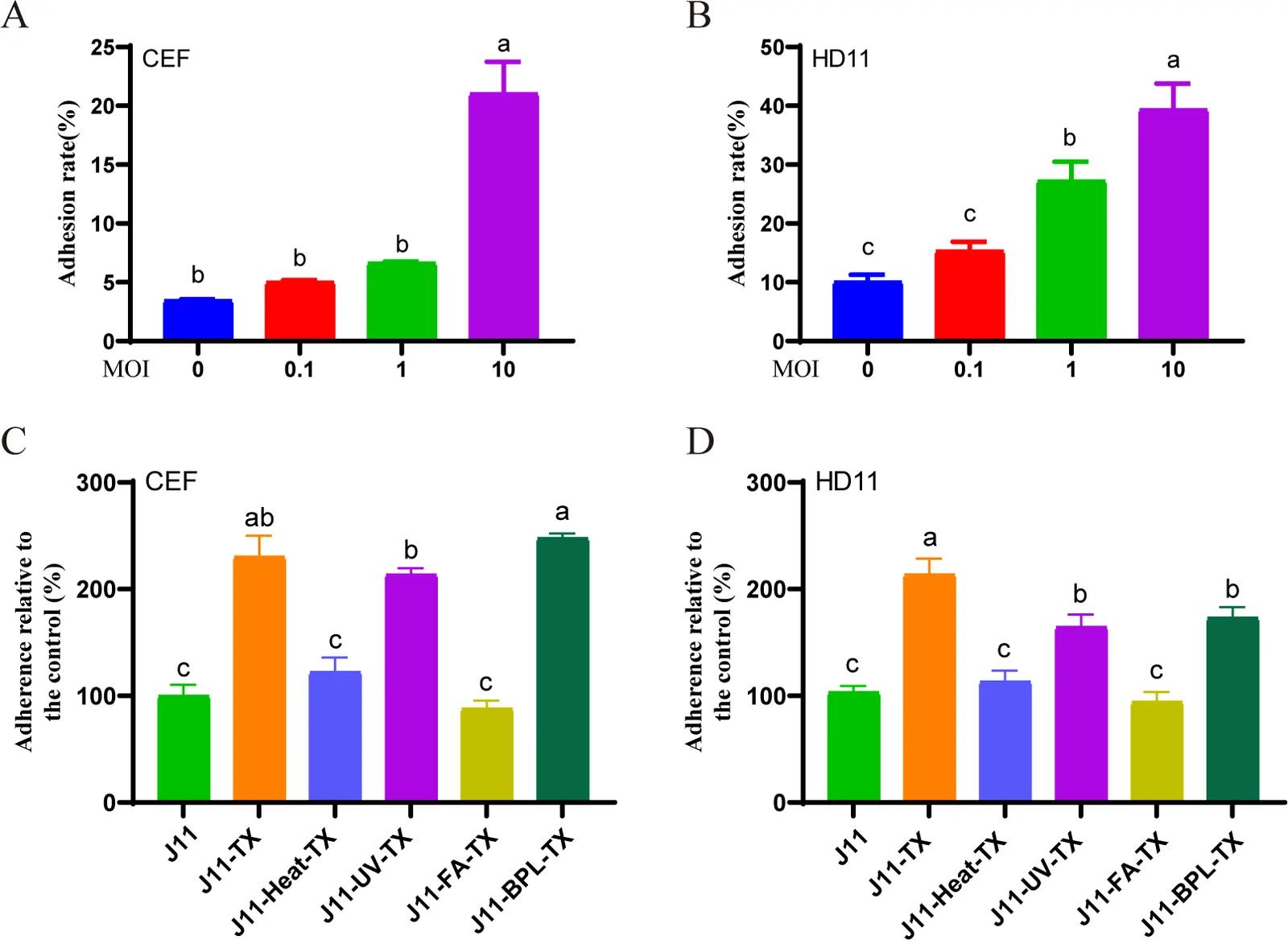
H9N2 AIV infection or co-incubation enhances *E. coli* adherence. CEF cells (**A**) and HD11 cells (**B**) were pre-infected with various MOIs of H9N2 AIV TX strain for 2 h, followed by inoculation with *E. coli* J11 strain at an MOI of 100. After incubation for 2 h, cells were lysed and cell-associated bacteria were recovered. The adhesion rate was calculated as a percentage of the inoculum. J11 and TX were co-cultured in CEF cells (**C**) and HD11 cells (**D**). The H9N2 AIV was inactivated by heat treatment, UV irradiation, formaldehyde treatment, or β-propiolactone treatment, respectively. The adhesion ratio was calculated by comparing the co-incubation groups to the J11 alone group. Data are presented as means ± SEM of three independent experiments. Different lowercase letters indicate significant differences (*P* < 0.05).

To determine the effect of H9N2 AIV and APEC co-infection on bacterial adhesion, we examined bacterial adherence to CEF and HD11 cells that were either infected or uninfected with AIV. The number of J11 adherents was positively correlated with the MOI of the TX virus (Fig. 1A and B). Co-incubation of TX and J11 resulted in a significant increase in bacterial adhesion compared to infection with J11 alone (Fig. 1C and D). Diverse methods have been employed to treat the virus when using inactivated TX strain for co-incubation. The β-propiolactone- or UV-inactivated TX strain effectively preserved HA activity, whereas heat- or formalin-inactivated TX strain resulted in a loss of HA activity (Table 3). Increased bacterial adhesion was observed after co-incubation with β-propiolactone or UV-inactivated TX strain, rather than heat- or formalin-inactivated TX strain, suggesting that intact viral hemagglutination activity may be required for the synergistic effect on adhesion. These data suggest that H9N2 AIV infection or co-incubation enhances APEC bacterial adhesion.

**TABLE 3:** Biological properties of H9N2 AIVs

Viruses	HA titer (log_2_）	Titer (log_10_TCID_50_/mL)
TX	9	5
Heat-TX	0	0
UV-TX	6	0
FA-TX	0	0
BPL-TX	6	0

### H9N2 AIV binds directly to the surface of APEC

**Fig 2:**
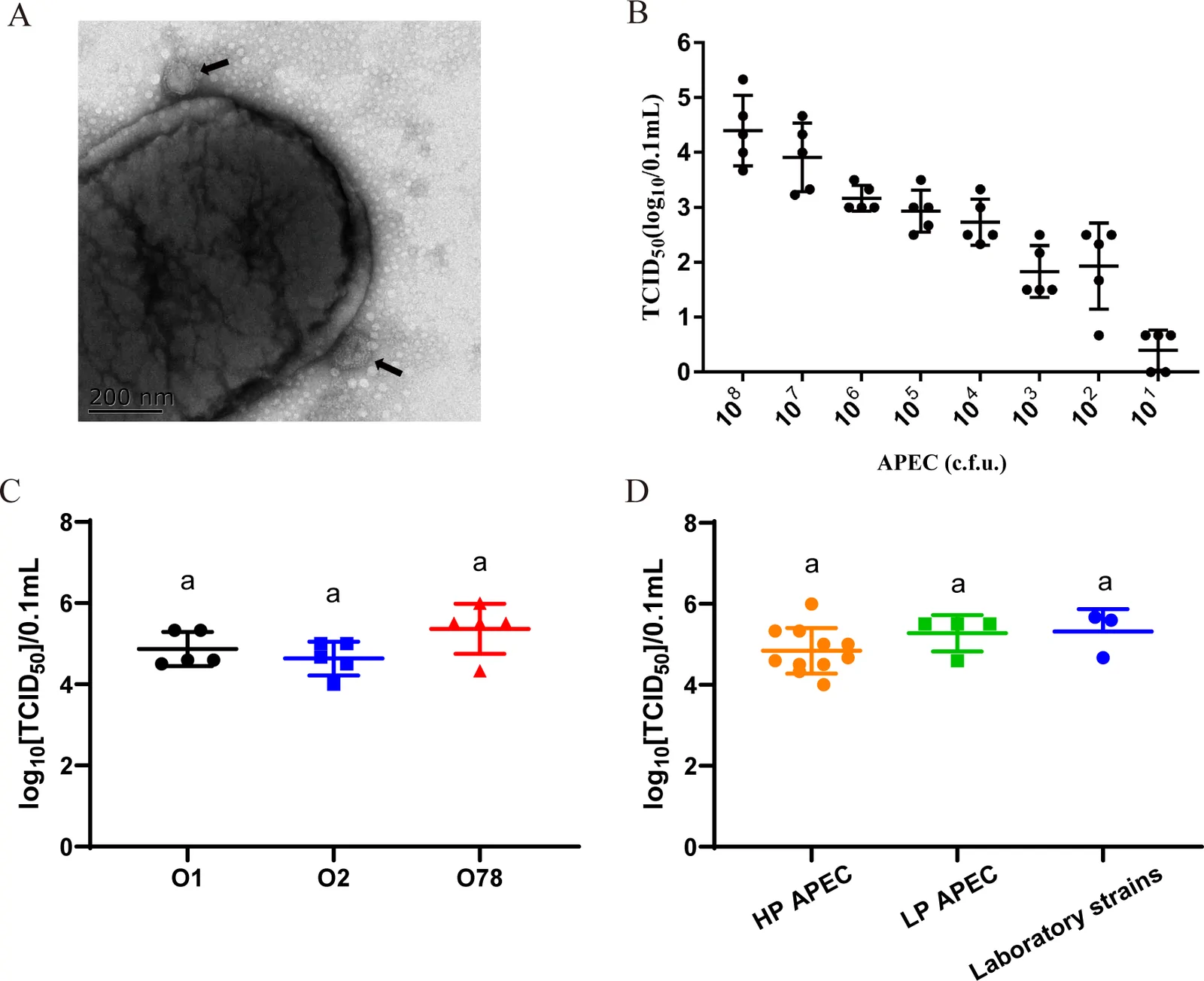
H9N2 AIV binds directly to various *E. coli* strains. H9N2 AIV and APEC strain J11 were mixed and incubated at 37°C for 30 min; the co-sedimented APEC and AIV particles were visualized by negative staining and imaging under electron microscopy; scale bars: 200 nm (**A**). Quantification of H9N2 AIV load co-sedimented with APEC was determined by TCID_50_, using different concentrations of APEC J11 strain (**B**), different serotypes of APEC strains (**C**), and *E. coli* strains with diverse pathogenicity levels (**D**). The *E. coli* strains were categorized into three groups: HP APEC, LP APEC, and laboratory strains (DH5α, BL21, Top10). Data are expressed as the mean ± SEM (*n* = 5) of three independent experiments. Different lowercase letters indicate significant differences (*P* < 0.05).

To evaluate the interaction between H9N2 AIV and APEC, purified TX was mixed with J11 and incubated for 30 min, after which the bacteria were collected by centrifugation. Observation using electron microscopy showed that the virus particles were directly bound to the surface of J11 (Fig. 2A). Quantification of the influenza virus bound to J11 was performed using TCID_50_, and the results showed that there was a direct correlation between the reduction in bacterial population and the corresponding decrease in viral load (Fig. 2B). To evaluate whether the binding between H9N2 AIV and *E. coli* is serotype- or pathogenicity-dependent, we first screened a panel of 18 *E. coli* strains (Table 1). These strains included O1, O2, and O78 APEC isolates (five strains for each serotype), as well as the laboratory strains DH5α, BL21, and Top10. Among the field isolates, strains carrying ≥2 virulence genes were classified as highly pathogenic APEC (HP APEC), while those carrying <2 virulence genes were considered low-pathogenic APEC (LP APEC). The results showed no significant difference in viral load among the three APEC serotypes (Fig. 2C). In addition, there was no significant difference in viral load among highly pathogenic APEC, low-pathogenic APEC, and the laboratory strains (Fig. 2D). These data suggest that H9N2 AIV can bind to the surface of different *E. coli* strains.

### APEC and H9N2 AIV complex further enhances bacterial adhesion

**Fig 3:**
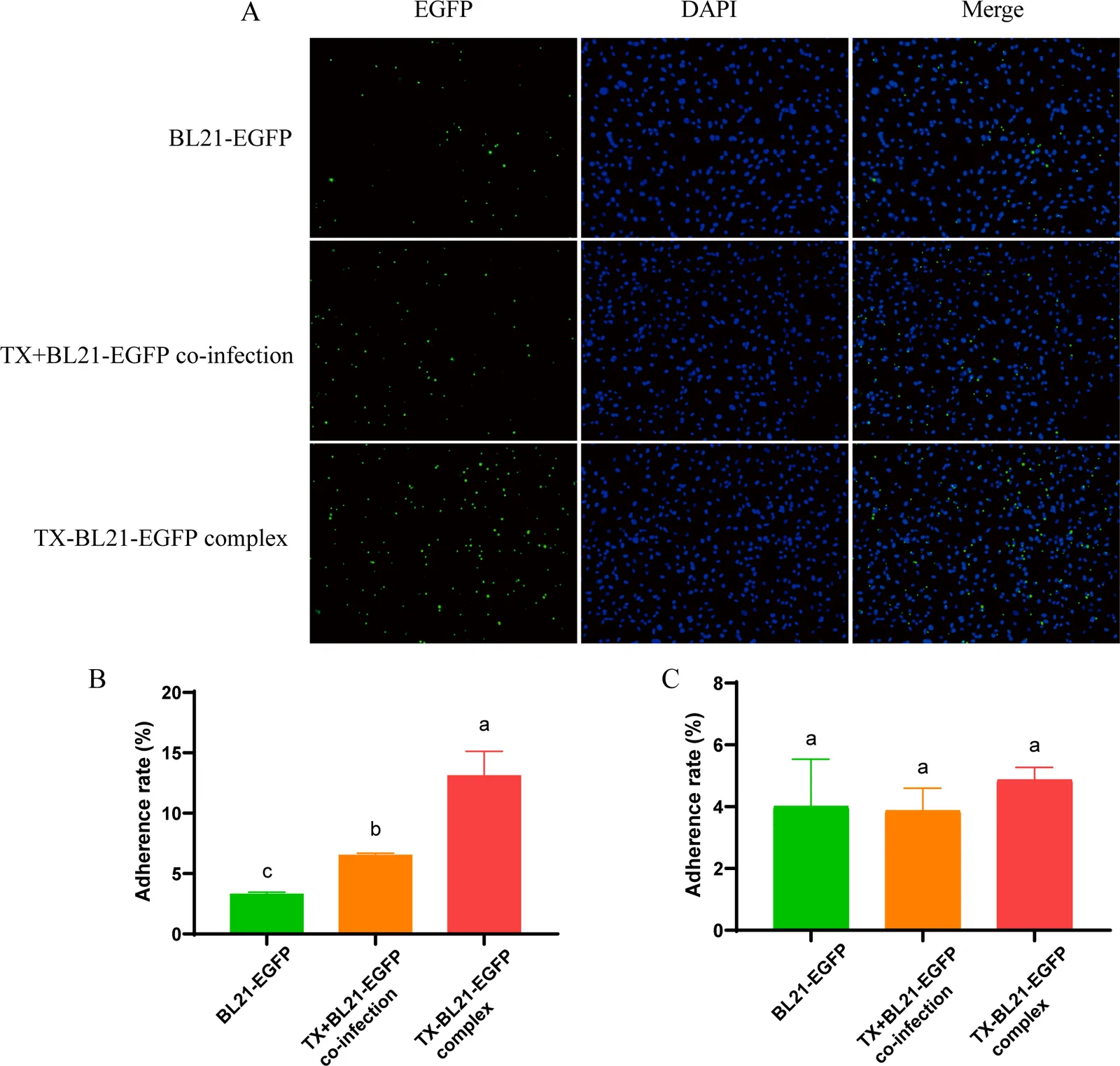
The complex of TX and BL21-EGFP enhances bacterial adhesion. 10⁸ CFU of BL21-EGFP alone, TX+BL21-EGFP co-infection (BL21-EGFP mixed with TX without pre-incubation), or TX-BL21-EGFP complex (BL21-EGFP pre-incubated with 10⁴·⁵ TCID₅₀ of TX for 30 min) were inoculated into CEF cells. Cells were stained with DAPI and observed under fluorescence microscopy (200×) (**A**). The rate of bacterial adhesion to CEF cells without (**B**) or with (**C**) treatment with antiserum against TX was calculated as a percentage of the initial inoculum. Data are presented as means ± SEM of three independent experiments. Different lowercase letters indicate significant differences (*P* < 0.05).

To compare the adhesion efficacy of *E. coli* to CEF cells, considering that H9N2 AIV exhibits a similar binding pattern to APEC and the laboratory *E. coli* strains, *E. coli* BL21(DE3) harboring an EGFP plasmid was used instead of wild-type APEC for direct visualization and quantification of bacterial adhesion. Three groups were set as follows: BL21-EGFP alone, the TX+BL21-EGFP co-infection (BL21-EGFP mixed with TX without pre-incubation), and the TX-BL21-EGFP complex (BL21-EGFP pre-incubated with TX for 30 min). The mixtures were inoculated into CEF cells. TX enhanced BL21-EGFP adhesion to CEF cells under both conditions (Fig. 3A). Interestingly, bacterial adhesion was further enhanced in the TX-BL21-EGFP complex group compared with the co-infection group (Fig. 3B). However, the enhanced adhesion was blocked by treatment with antiserum against TX (Fig. 3C).

### Pathogenicity effects of co-infection and complex infection of APEC and TX

**Fig 4:**
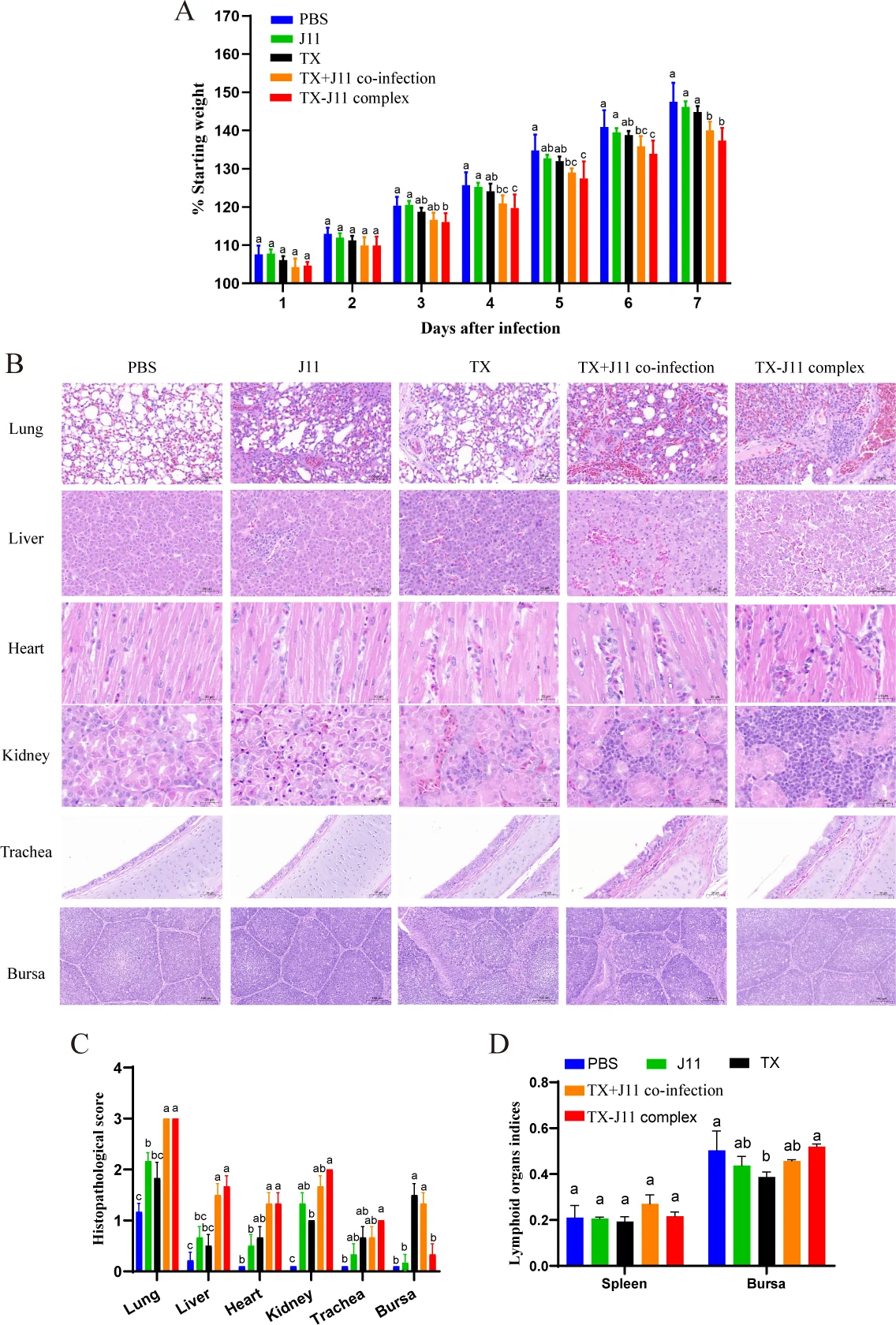
Complex or co-infection with *E. coli* and H9N2 AIV reduces body weight gain and exacerbates histopathological lesions. Three-week-old SPF chickens were randomly divided into five groups: PBS control, *E. coli* J11 alone, TX alone, TX+J11 co-infection, and TX-J11 complex infection, and monitored for 7 days. (**A**) Body weight changes in each group (*n* = 5 per group). (**B**) Representative histopathological lesions visualized by H&E staining. Scale bars: 20 µm, 50 µm, or 100 µm. (**C**) Histopathological scores (*n* = 3 per group). (**D**) Lymphoid organ index expressed as (organ weight/body weight) × 100%. Data are presented as means ± SEM. Statistical significance was assessed by two-way ANOVA to compare results among groups. Different lowercase letters indicate significant differences (*P* < 0.05).

To evaluate the effects of direct virus-bacteria interactions *in vivo*, J11 was co-incubated or pre-incubated with TX, and the mixtures were administered to chickens by intranasal inoculation. The results showed that body weight gain in the TX-J11 complex group was significantly lower than that in the PBS group from 3 to 7 dpi, while the TX+J11 co-infection group showed significantly lower body weight gain than the PBS group from 4 to 7 dpi. No significant differences were observed between the PBS group and either the TX or J11 group throughout the experiment (Fig. 4A). Histopathological analysis revealed varying degrees of damage across different organs. Compared with the PBS group, the J11 group showed significantly higher scores in the lungs and kidneys; the TX group showed significantly higher scores in the kidneys and bursa; the TX+J11 co-infection group showed significantly higher scores in the lungs, liver, heart, kidneys, and bursa; and the TX-J11 complex group showed significantly higher scores in the lungs, liver, heart, trachea, and kidneys (Fig. 4C). However, the bursa in the TX and TX+J11 co-infection groups displayed atrophy of the lymphatic follicles and hyperplasia of interstitial fibers around the follicles (Fig. 4B). Furthermore, the lymphoid organ indices indicated a lower bursa index in the TX group than in the control and other groups, along with normal splenic development (Fig. 4D). These data indicate that the pathogenicity of TX+J11 co-infection and TX-J11 complex infection in chickens is higher than that of single infections.

**Fig 5:**
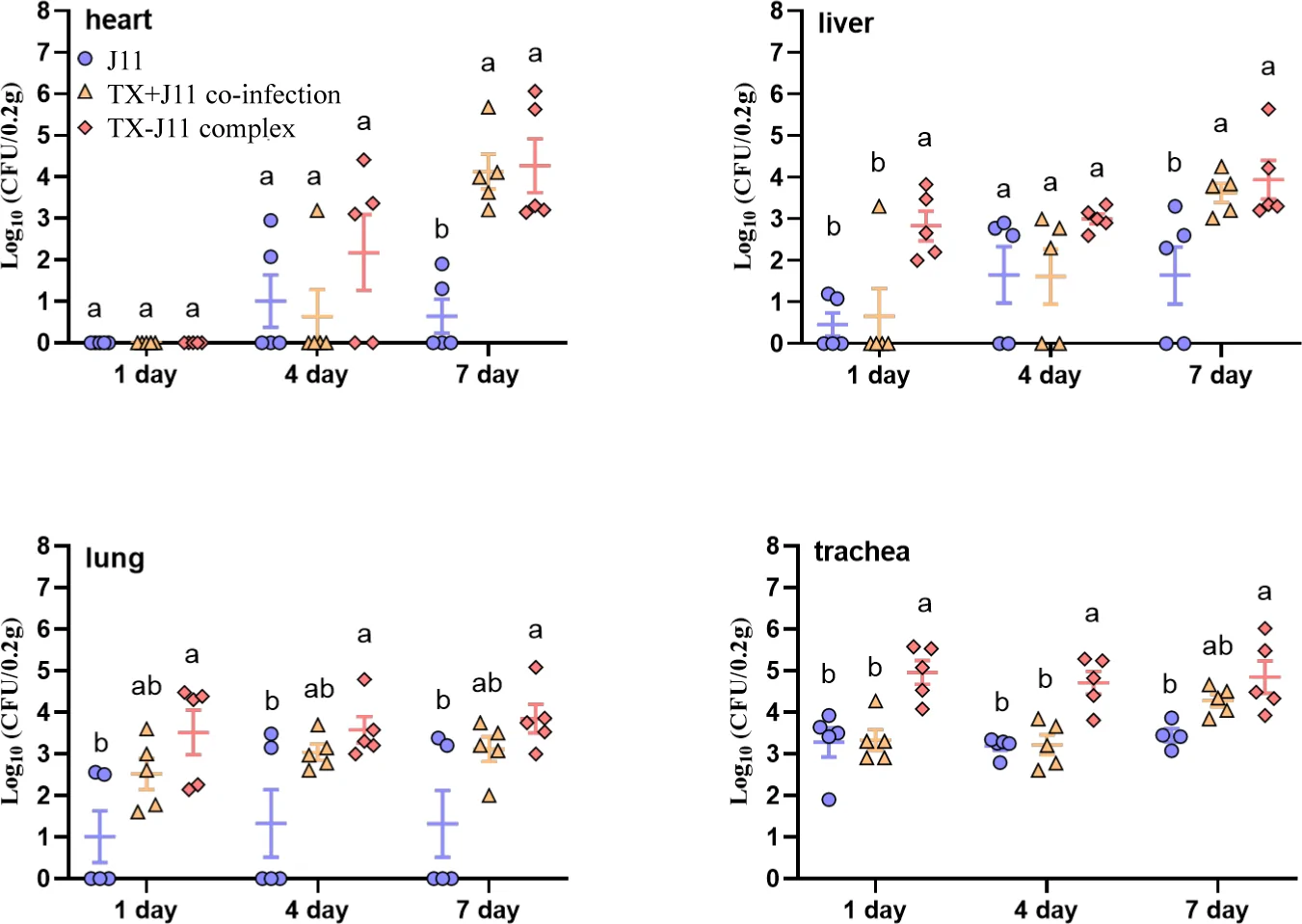
TX-J11 complex infection increases bacterial loads in organs. Chickens from each group were euthanized at 1, 4, and 7 dpi. The heart, liver, lungs, and trachea were collected for bacterial quantification. Each point represents an individual sample, and bars indicate the median (*n* = 5 chickens per group). Data are presented as means ± SEM. Statistical significance was assessed by two-way ANOVA to compare results among groups. Different lowercase letters indicate significant differences (*P* < 0.05).

Chickens were euthanized at 1, 4, and 7 dpi, and bacterial loads in the heart, liver, lungs, and tracheal lavage were determined. Compared with the J11 group, both the TX+J11 co-infection and TX-J11 complex groups showed significantly higher bacterial loads in the heart and liver at 7 dpi; additionally, the complex group also showed significantly higher loads in the liver at 1 dpi. In the lungs and tracheal lavage fluid, the TX-J11 complex group exhibited significantly higher bacterial loads than the J11 group at all three time points, whereas no significant differences were observed in the co-infection group at any time point (Fig. 5). This suggests that J11 and TX complex infections increase bacterial colonization *in vivo*.

**Fig 6:**
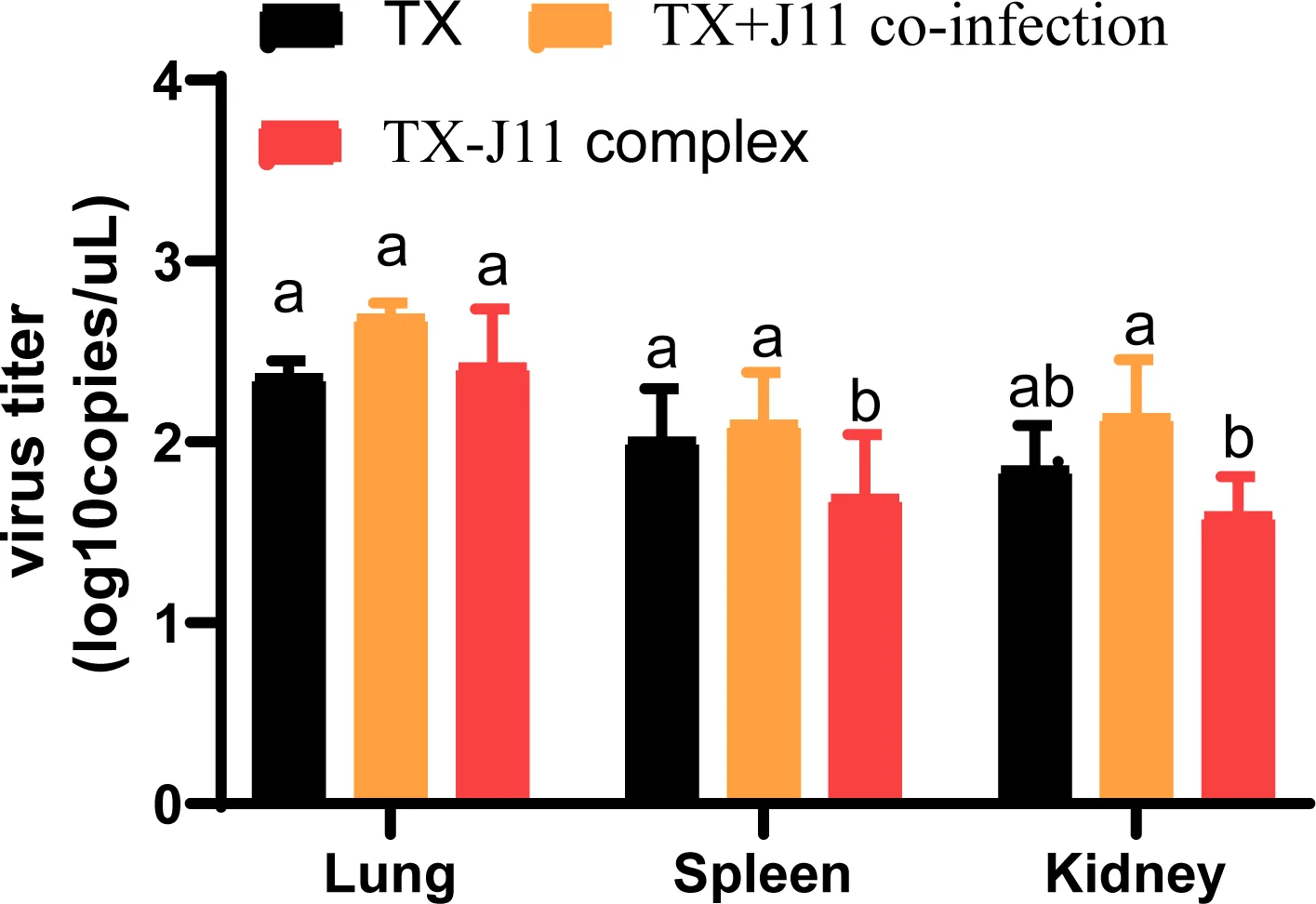
TX-J11 complex infection inhibits viral replication. Viral loads were determined in the lungs, spleen, and kidneys at 4 dpi (*n* = 5 chickens per group). Data are presented as means ± SEM. Different lowercase letters indicate significant differences (*P* < 0.05).

Absolute quantitative PCR analysis was used to determine the viral loads of infected chickens, with 10 chickens in each experimental group and 5 in the PBS group. Compared with chickens infected with TX alone, the TX+J11 co-infection showed a higher number of positive chickens in both throat and cloaca samples at 1 and 4 dpi, whereas no significant difference was observed between the TX and TX-J11 complex groups (Table 4). This experiment was performed once with 10 chickens per group in the experimental groups and 5 chickens in the PBS group. Moreover, the viral loads of the spleen in the TX-J11 complex group significantly decreased (Fig. 6). These findings indicate that TX-J11 complex infection inhibited TX replication *in vivo*.

**TABLE 4:** Detection of influenza virus in throat and cloaca samples by quantitative PCR

Group	1 dpi		4 dpi	
	Throat	Cloaca	Throat	Cloaca
TX	5/10	3/10	5/10	2/10
TX+J11 co-infection	8/10	5/10	8/10	4/10
TX-J11 complex	5/10	2/10	5/10	2/10
PBS	0/5	0/5	0/5	0/5

**Fig 7:**
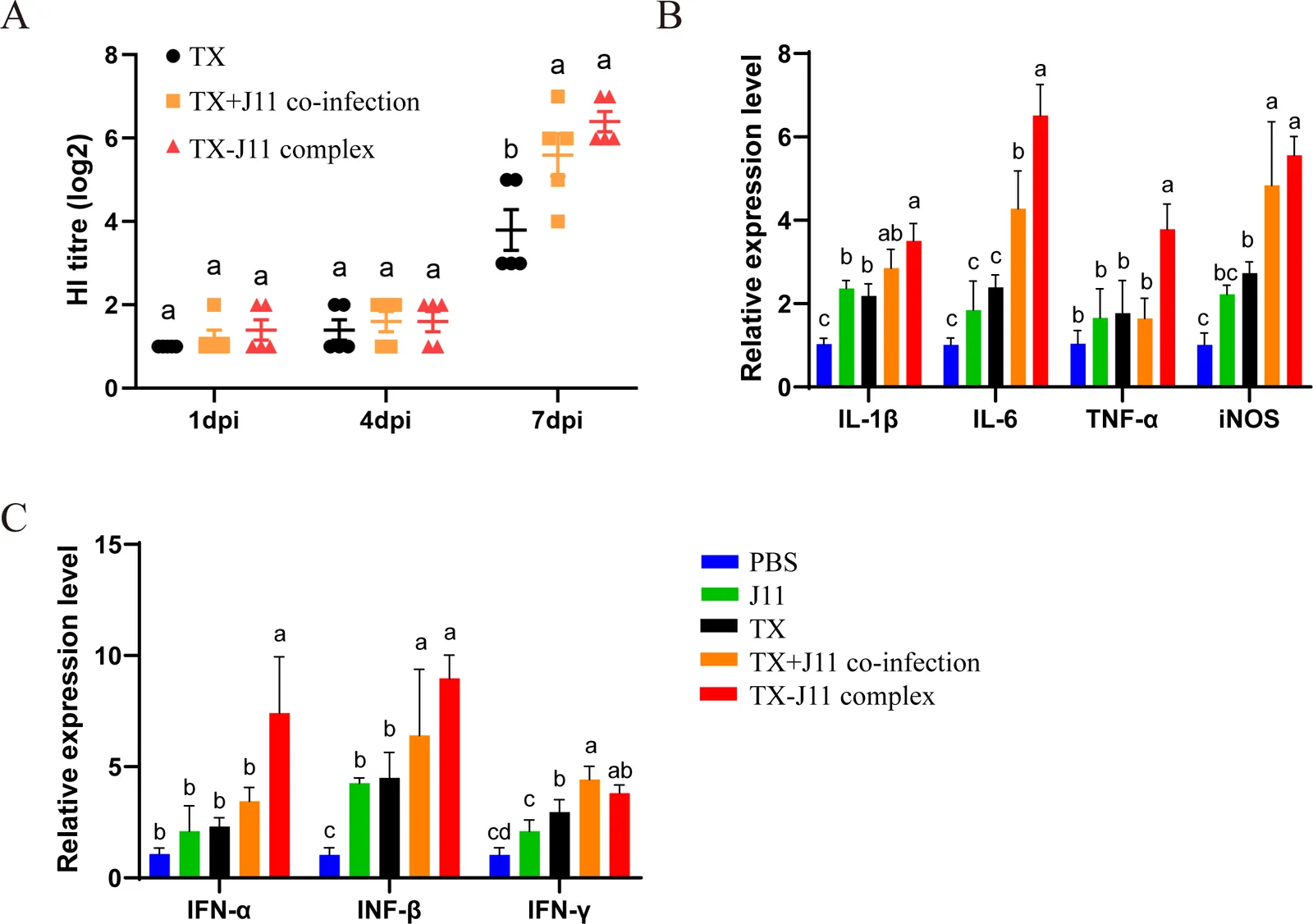
TX-J11 complex infection enhances immune responses in chickens. Five groups of SPF chickens were infected with J11 alone, TX alone, TX+J11 co-infection, or TX-J11 complex. (**A**) Serum HI antibody titers were measured at 1, 4, and 7 dpi. (**B**) Expression levels of pro-inflammatory factors IL-1β, IL-6, TNF-α, and iNOS, and (**C**) interferons in chicken lungs were detected by real-time quantitative PCR at 7 dpi. Data are expressed as mean mRNA expression normalized to β-actin (*n* = 3). Data are presented as means ± SEM. Different lowercase letters indicate significant differences (*P* < 0.05).

Serum HI antibody titers were assessed, and a significant increase in antibody levels was observed at 7 dpi in the TX+J11 co-infection group as well as in the TX-J11 complex group (Fig. 7A). Subsequently, the mRNA expression levels of the pro-inflammatory factors IL-1β, IL-6, TNF-α, and iNOS in the lung tissues were analyzed at 7 dpi. The mRNA expression levels of pro-inflammatory factors were significantly higher in the TX-J11 complex groups than in the TX and J11 single-infection groups (Fig. 7B). Additionally, the mRNA expression levels of type I, type II, and type III interferons were measured. While IFN-β and IFN-γ expression increased in the TX+J11 co-infection group compared to that in the TX and J11 alone groups, significant increases in IFN-α and IFN-β were observed in the TX-J11 complex group. These findings indicate that co-infection and complex infection of TX and J11 enhance both antibody and innate immune responses (Fig. 7C).

### APEC reduces the thermal stability of viruses by altering their particle morphology in the complex

**Fig 8:**
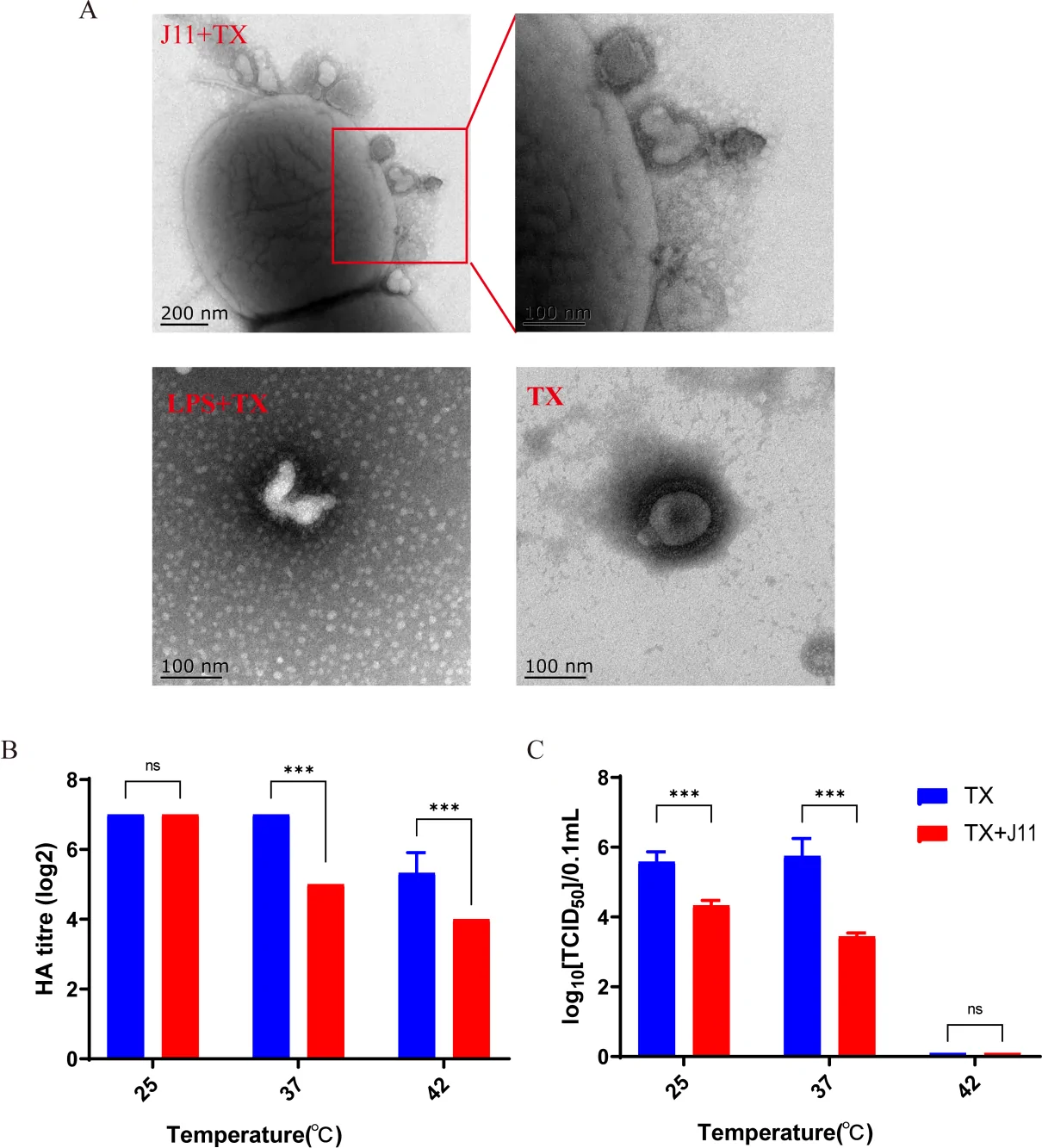
Co-incubation with J11 impaired the morphology and thermostability of viral particles. (**A**) Morphology of TX and J11 after incubation at 37°C for 3 h was observed under transmission electron microscopy. The virus particles with disrupted vesicle membrane structure were highlighted in the red outline. Bars: 100 nm or 200 nm. HA titers (**B**) and TCID_50_ (**C**) of TX virus after incubation with J11 at 25°C, 37°C, and 42°C for 3 h, respectively. Statistical significance is assessed by two-way ANOVA to compare the results among different groups. Data are presented as means ± SEM of three independent experiments. *P < 0.05; **P < 0.01; ***P < 0.001.

Since the influenza virus inhibits virus replication *in vivo* after binding to the surface of APEC, we next explored the effect of this long-term direct action on the virus. TX was incubated with J11 at 37°C for 3 h. Electron microscopy observations showed that the morphology of the virus particles was altered, leading to the destruction and deformation of their original vesicle structure (Fig. 8A), similar to the morphology observed during LPS and TX co-incubation. The HA titer and viral activity of the virus were examined at different temperatures. The results showed that the viral HA titers and infectivity were significantly decreased when co-incubated with J11 for 3 h at 37°C and 42°C. Moreover, prolonged incubation with J11 can affect viral thermal stability by altering the structural integrity of vesicle membranes (Fig. 8B and C).

The mixtures of TX with J11 after 3 h incubation at 37°C were inoculated intranasally into SPF chickens, with three chickens in each group, and the viral shedding of infected chickens was continuously monitored. The results showed that the chickens in the TX-infected group continued to shed in throat samples at 1 and 4 dpi. However, no viral shedding was detected in the TX-J11 complex group (Table 5). This result indicated that viral infectivity was inhibited by co-incubation with J11.

**TABLE 5:** Influenza virus detection under prolonged incubation by qPCR

Group	1 dpi		4 dpi	
Time	Throat	Cloaca	Throat	Cloaca
TX	3/3	0/3	2/3	0/3
TX-J11 complex	0/3	0/3	0/3	0/3
PBS	0/3	0/3	0/3	0/3

## DISCUSSION

Secondary bacterial infections following H9N2 influenza virus infection are a common complication in poultry. During co-infection, an increase in bacterial load is a key step in morbidity or mortality (28). H9N2 AIV NS1 protein-mediated activation of the TGF-β signaling pathway upregulates the expression of fibronectin, leading to increased adhesion of APEC (17). The neuraminidase of influenza viruses has been shown to promote the cleavage of host salivary acids, which provides a source of nutrients for bacteria and thus promotes bacterial growth and reproduction (29). We found that the HA activity of the influenza virus may be important for the adhesion of *E. coli*. In the *in vitro* adhesion assay, the enhancement of bacterial adhesion by H9N2 AIV was more pronounced in HD11 macrophages than in CEF fibroblasts, especially at lower MOIs. This difference may be attributed to the distinct biological properties of these two cell types. HD11 cells are professional phagocytes that express a broader range of pattern recognition receptors and are more permissive to viral entry, which may facilitate virus-bacteria interactions at the cell surface. In contrast, CEF cells are adherent epithelial-like cells with a more limited capacity for viral uptake and a different surface glycoprotein profile, which may require a higher viral dose to achieve a similar effect. Nonetheless, both cell types consistently showed a dose-dependent increase in bacterial adhesion, supporting the conclusion that viral presence promotes bacterial attachment. This enhancing effect was directed toward bacterial adhesion rather than bacterial growth, as there was no significant difference in the total bacterial count across all groups following inoculation into cells. Thus, an influenza virus with an intact envelope structure may be a prerequisite for its interaction with *E. coli*. Interestingly, we observed that H9N2 influenza virus particles were able to bind directly to both highly pathogenic and low-pathogenic APEC, with the distinction based on the presence of virulence genes. Although the bacterial ligands bound by H9N2 AIV remain unclear, we used H9N2 AIV and APEC complexes to examine their effects on virus-mediated bacterial infections. Our research showed that the complexes were able to enhance APEC adhesion *in vitro* and increase bacterial colonization *in vivo*. Notably, the loss of HA activity after heat or formalin inactivation abolished this effect, suggesting that intact HA may play a role in mediating bacterial adhesion, although the precise mechanism remains to be elucidated.

The effects of bacteria on viruses at the same infection site may be synergistic or antagonistic, and these interactions in turn influence the severity of the disease complex. A prior condition of influenza virus infection is the activation of HA; in addition to host cell proteases, bacterial proteases can be used directly for HA activation by low-pathogenic AIV (30). Some *Staphylococcus* sp. are able to promote HA cleavage of H9N2 AIV using the virulence factor staphylococcal kinase, which activates plasminogen (31). Conversely, pre-infection with *Mycoplasma gallisepticum* inhibits AIV infection by reducing the number of H9N2 receptors, inducing cilia destruction, epithelial cell proliferation, and modulation of the innate immune response (32). Our study showed that the interaction of these complexes resulted in a significant increase in the bacterial load and a significant decrease in the viral load compared to single infections or co-infections. Notably, H9N2 AIV caused atrophy and damage to the bursa, leading to a decrease in the bursal index, while no changes were recorded in the complexes. Our results are consistent with a previous study showing that H9N2 AIV leads to immunosuppression by damaging immune organs, thus increasing disease severity (33). In addition, complexes and co-infections significantly increased the level of antibody response in chickens, which may be influenced by two factors: the inflammatory response and adjuvant effect of LPS in *E. coli*. It has been previously shown that the prophylactic use of LPS reduces the viral load in macrophages of chickens infected with low-pathogenic AIV (34). In addition, pre-stimulation of Toll-like receptors 2 and 4 enhances resistance to highly pathogenic influenza viruses in mice (35). Our results show that the complex and co-infection caused upregulation of mRNA expression of lung inflammatory factors (IL-1β, IL-6, IFN-α, and iNOS) and type I, II, and III IFN. This may be related to the reduction in the viral load in the organ by the complex.

Bacteria and bacterial components have complex effects on the stability and infectivity of viral particles, varying depending on the viral species, subtype, and host origin. In the gastrointestinal tract, bacteria and their cytosolic components, LPS and peptidoglycan, enhance mammalian reovirus thermostability through interactions with viral particles, which increases reovirus infection in the intestine (36). Direct interactions between influenza A virus (IAV) and the polysaccharide capsule of specific human upper respiratory tract bacteria, including *S. pneumoniae* and *Haemophilus influenzae*, have been found to promote the stability and infectivity of IAV during desiccation stress (20). However, the interaction of human H1N1 PR8 influenza viruses with LPS reduces the long-term persistence and stability of the viruses, whereas avian H3N8 viruses are not significantly affected (37). In our study, H9N2 AIV complexes with APEC showed a trend toward decreased tissue viral load and reduced viral detection in laryngeal and cloacal samples. Interestingly, we also found that APEC reduced the thermal stability of AIV by altering its vesicle membrane structure. For the *in vivo* thermal stability experiment, a limited number of chickens (*n* = 3 per group) was used as a pilot study to assess whether the observed *in vitro* effects on viral morphology and infectivity could translate *in vivo*. Despite the modest sample size, the results consistently showed no viral detection in the complex group, supporting the *in vitro* findings. Future studies with larger cohorts will be valuable to further validate these observations. Additionally, the two infection models used in this study—co-infection (simultaneous inoculation) and complex infection (pre-incubated virus-bacteria mixture)—were designed to distinguish between the effects of independent viral and bacterial co-exposure versus direct physical interaction prior to host entry. The complex infection model allowed us to specifically evaluate the consequences of preformed virus-bacteria complexes, which may represent an early stage of co-infection where direct binding occurs before reaching the host epithelium. Together, these findings highlight the importance of direct virus-bacteria interactions in shaping infection outcomes and provide a basis for further mechanistic studies.

### Conclusion

In this study, we explored the direct interaction between H9N2 AIV and APEC and demonstrated that TX can bind directly to the surface of APEC, further promoting its adherence. This suggests that APEC can exploit the influenza virus as a mediator to enhance host adherence and infection, leading to increased inflammatory responses and exacerbated lung damage. These findings emphasize the complexity of the pathogenesis of both H9N2 AIV and APEC and may represent a new strategy to explore APEC infection in hosts.

## Data Availability

The data generated during the study are available at HARVARD Dataverse name (“Figure raw data”) at https://doi.org/10.7910/DVN/OBYZGU.

## References

[1] Gu M, Xu L, Wang X, Liu X. 2017. Current situation of H9N2 subtype avian influenza in China. Vet Res 48:49. doi:10.1186/s13567-017-0453-2 28915920 PMC5603032

[2] Peacock THP, James J, Sealy JE, Iqbal M. 2019. A global perspective on H9N2 Avian influenza virus. Viruses 11:620. doi:10.3390/v11070620 31284485 PMC6669617

[3] Su S, Bi Y, Wong G, Gray GC, Gao GF, Li S. 2015. Epidemiology, evolution, and recent outbreaks of Avian influenza virus in China. J Virol 89:8671–8676. doi:10.1128/JVI.01034-15 26063419 PMC4524075

[4] Pu J, Yin Y, Liu J, Wang X, Zhou Y, Wang Z, Sun Y, Sun H, Li F, Song J, et al.. 2021. Reassortment with dominant chicken H9N2 influenza virus contributed to the fifth H7N9 virus human epidemic. J Virol 95:e01578-20. doi:10.1128/JVI.01578-20 33731452 PMC8139711

[5] Pan Q, Liu A, Zhang F, Ling Y, Ou C, Hou N, He C. 2012. Co-infection of broilers with Ornithobacterium rhinotracheale and H9N2 avian influenza virus. BMC Vet Res 8:104. doi:10.1186/1746-6148-8-104 22748160 PMC3424113

[6] Sun Y, Liu J. 2015. H9N2 influenza virus in China: a cause of concern. Protein Cell 6:18–25. doi:10.1007/s13238-014-0111-7 25384439 PMC4286136

[7] Arafat N, Abd El Rahman S, Naguib D, El-Shafei RA, Abdo W, Eladl AH. 2020. Co-infection of *Salmonella enteritidis* with H9N2 avian influenza virus in chickens. Avian Pathol 49:496–506. doi:10.1080/03079457.2020.1778162 32835500

[8] Bo-shun Z, Li L, Qian Z, Zhen W, Peng Y, Guo-dong Z, Wen-jian S, Xue-fei C, Jiang S, Zhi-jing X. 2020. Co-infection of H9N2 influenza virus and *Pseudomonas aeruginosa* contributes to the development of hemorrhagic pneumonia in mink. Vet Microbiol 240:108542. doi:10.1016/j.vetmic.2019.108542 31902499

[9] Dong J, Zhou Y, Pu J, Liu L. 2022. Status and challenges for vaccination against Avian H9N2 influenza virus in China. Life (Basel) 12:1326. doi:10.3390/life12091326 36143363 PMC9505450

[10] Liu S, Zhuang Q, Wang S, Jiang W, Jin J, Peng C, Hou G, Li J, Yu J, Yu X, Liu H, Sun S, Yuan L, Chen J. 2020. Control of avian influenza in China: strategies and lessons. Transbound Emerg Dis 67:1463–1471. doi:10.1111/tbed.13515 32065513

[11] Hu J, Afayibo DJA, Zhang B, Zhu H, Yao L, Guo W, Wang X, Wang Z, Wang D, Peng H, Tian M, Qi J, Wang S. 2022. Characteristics, pathogenic mechanism, zoonotic potential, drug resistance, and prevention of avian pathogenic Escherichia coli (APEC). Front Microbiol 13:1049391. doi:10.3389/fmicb.2022.1049391 36583051 PMC9793750

[12] Nawaz S, Wang Z, Zhang Y, Jia Y, Jiang W, Chen Z, Yin H, Huang C, Han X. 2024. Avian pathogenic *Escherichia coli* (APEC): current insights and future challenges. Poult Sci 103:104359. doi:10.1016/j.psj.2024.104359 39388979 PMC11490931

[13] Wang J, Li Y, Yin Y. 2018. Respiratory phagocytes are implicated in enhanced colibacillosis in chickens co-infected with influenza virus H9N2 and *Escherichia coli*. Br Poult Sci 59:160–165. doi:10.1080/00071668.2017.1406061 29148834

[14] Abd El-Hack ME, El-Saadony MT, Alqhtani AH, Swelum AA, Salem HM, Elbestawy AR, Noreldin AE, Babalghith AO, Khafaga AF, Hassan MI, El-Tarabily KA. 2022. The relationship among avian influenza, gut microbiota and chicken immunity: an updated overview. Poult Sci 101:102021. doi:10.1016/j.psj.2022.102021 35939896 PMC9386105

[15] Zhang X, Zhao Q, Wu C, Xie Z, Ci X, Li H, Lin W, Zhang H, Xie Q. 2020. Nitrate is crucial for the proliferation of gut *Escherichia coli* caused by H9N2 AIV infection and effective regulation by Chinese herbal medicine ageratum-liquid. Front Microbiol 11:555739. doi:10.3389/fmicb.2020.555739 33193136 PMC7662154

[16] Wang X, Wang H, Zhang S, Shang H, Wang C, Zhou F, Gao P, Zhu R, Hu L, Wei K. 2023. The role of transforming growth factor beta-1 protein in *Escherichia coli* secondary infection induced by H9N2 avian influenza virus in chickens. Microb Pathog 175:105983. doi:10.1016/j.micpath.2023.105983 36641002

[17] Han J, Chang W, Fang J, Hou X, Li Z, Wang J, Deng W. 2024. The H9N2 avian influenza virus increases APEC adhesion to oviduct epithelia by viral NS1 protein-mediated activation of the TGF-β pathway. J Virol 98:e0151223. doi:10.1128/jvi.01512-23 38415626 PMC10949501

[18] Wang Y, Gagnon CA, Savard C, Music N, Srednik M, Segura M, Lachance C, Bellehumeur C, Gottschalk M. 2013. Capsular sialic acid of Streptococcus suis serotype 2 binds to swine influenza virus and enhances bacterial interactions with virus-infected tracheal epithelial cells. Infect Immun 81:4498–4508. doi:10.1128/IAI.00818-13 24082069 PMC3837972

[19] Rowe HM, Meliopoulos VA, Iverson A, Bomme P, Schultz-Cherry S, Rosch JW. 2019. Direct interactions with influenza promote bacterial adherence during respiratory infections. Nat Microbiol 4:1328–1336. doi:10.1038/s41564-019-0447-0 31110359 PMC7069060

[20] Rowe HM, Livingston B, Margolis E, Davis A, Meliopoulos VA, Echlin H, Schultz-Cherry S, Rosch JW. 2020. Respiratory bacteria stabilize and promote airborne transmission of influenza a virus. mSystems 5:e00762-20. doi:10.1128/mSystems.00762-20 32873612 PMC7470989

[21] French AJ, Rockey NC, Le Sage VL, et al.. 2023. Detection of influenza virus and *Streptococcus pneumoniae* in air sampled from co-infected ferrets and analysis of their influence on pathogen stability. mSphere 8:e0003923. doi:10.1101/2023.02.24.529988 37255295 PMC10449498

[22] Wang S, Meng Q, Dai J, Han X, Han Y, Ding C, Liu H, Yu S. 2014. Development of an allele-specific PCR assay for simultaneous sero-typing of avian pathogenic *Escherichia coli* predominant O1, O2, O18 and O78 strains. PLoS One 9:e96904. doi:10.1371/journal.pone.0096904 24805368 PMC4013041

[23] Schierack P, Steinrück H, Kleta S, Vahjen W. 2006. Virulence factor gene profiles of *Escherichia coli* isolates from clinically healthy pigs. Appl Environ Microbiol 72:6680–6686. doi:10.1128/AEM.02952-05 17021219 PMC1610323

[24] Johnson TJ, Wannemuehler Y, Doetkott C, et al.. 2008. Identification of minimal predictors of avian pathogenic *Escherichia coli* virulence for use as a rapid diagnostic tool. J Clin Microbiol 46:3987–3996. doi:10.1128/JCM.00816-08 18842938 PMC2593276

[25] Zhu Y, Yang D, Ren Q, Yang Y, Liu X, Xu X, Liu W, Chen S, Peng D, Liu X. 2015. Identification and characterization of a novel antigenic epitope in the hemagglutinin of the escape mutants of H9N2 avian influenza viruses. Vet Microbiol 178:144–149. doi:10.1016/j.vetmic.2015.04.012 25934533

[26] Spackman E. 2020. Animal influenza virus: methods and protocols. Third ed. Humana Press, New York.

[27] Purohit K, Solanki S, Singh G, Nagarajan SS. 2020. Assessment of experimental pathogenicity of avian influenza virus H9N2 isolates by intravenous pathogenicity index (IVPI) test and histopathology. Int J Chem Stud 8:764–770. doi:10.22271/chemi.2020.v8.i2l.8860

[28] Sarowska J, Futoma-Koloch B, Jama-Kmiecik A, Frej-Madrzak M, Ksiazczyk M, Bugla-Ploskonska G, Choroszy-Krol I. 2019. Virulence factors, prevalence and potential transmission of extraintestinal pathogenic *Escherichia coli* isolated from different sources: recent reports. Gut Pathog 11:10. doi:10.1186/s13099-019-0290-0 30828388 PMC6383261

[29] King S.J., Hippe K.R., Weiser J.N.. 2006. Deglycosylation of human glycoconjugates by the sequential activities of exoglycosidases expressed by *Streptococcus pneumoniae*. Mol Microbiol 59:961–974. doi:10.1111/j.1365-2958.2005.04984.x 16420364

[30] Böttcher-Friebertshäuser E, Klenk H.D., Garten W. 2013. Activation of influenza viruses by proteases from host cells and bacteria in the human airway epithelium. Pathog Dis 69:87–100. doi:10.1111/2049-632X.12053 23821437 PMC7108517

[31] Tse L.V., Whittaker G.R.. 2015. Modification of the hemagglutinin cleavage site allows indirect activation of avian influenza virus H9N2 by bacterial staphylokinase. Virology 482:1–8. doi:10.1016/j.virol.2015.03.023 25841078 PMC4461493

[32] Sid H, Hartmann S, Petersen H, Ryll M, Rautenschlein S. 2016. Mycoplasma gallisepticum modifies the pathogenesis of influenza A virus in the avian tracheal epithelium. Int J Med Microbiol 306:174–186. doi:10.1016/j.ijmm.2016.04.001 27079856

[33] Qiang F, Youxiang D. 2011. The effects of H9N2 influenza a on the immune system of broiler chickens in the Shandong Province. Transbound Emerg Dis 58:145–151. doi:10.1111/j.1865-1682.2010.01192.x 21205254

[34] St. Paul M, Mallick AI, Read LR, Villanueva AI, Parvizi P, Abdul-Careem MF, Nagy É, Sharif S. 2012. Prophylactic treatment with Toll-like receptor ligands enhances host immunity to avian influenza virus in chickens. Vaccine (Auckl) 30:4524–4531. doi:10.1016/j.vaccine.2012.04.033 22531557

[35] Shinya K, Okamura T, Sueta S, Kasai N, Tanaka M, Ginting TE, Makino A, Eisfeld AJ, Kawaoka Y. 2011. Toll-like receptor pre-stimulation protects mice against lethal infection with highly pathogenic influenza viruses. Virol J 8:97. doi:10.1186/1743-422X-8-97 21375734 PMC3061943

[36] Berger AK, Yi H, Kearns DB, Mainou BA. 2017. Bacteria and bacterial envelope components enhance mammalian reovirus thermostability. PLoS Pathog 13:e1006768. doi:10.1371/journal.ppat.1006768 29211815 PMC5734793

[37] Bandoro C, Runstadler JA. 2017. Bacterial lipopolysaccharide destabilizes influenza viruses. mSphere 2:e00267–e00317. doi:10.1128/mSphere.00267-17 29034326 PMC5636225

